# Enhanced flux pinning isotropy by tuned nanosized defect network in superconducting YBa_2_Cu_3_O_6+x_ films

**DOI:** 10.1038/s41598-019-51978-0

**Published:** 2019-10-28

**Authors:** Mukarram Zaman Khan, Elmeri Rivasto, Jussi Tikkanen, Hannes Rijckaert, Mika Malmivirta, Maciej Oskar Liedke, Maik Butterling, Andreas Wagner, Hannu Huhtinen, Isabel Van Driessche, Petriina Paturi

**Affiliations:** 1 Wihuri Physical Laboratory, Department of Physics and Astronomy, University of Turku, FI-20014 Turku, Finland; 2 University of Turku Graduate School (UTUGS), University of Turku, FI-20014 Turku, Finland; 3 SCRiPTS, Department of Chemistry, Ghent University, Krijgslaan 281 S3, 9000 Ghent, Belgium; 4 Institute of Radiation Physics, Helmholtz-Zentrum Dresden - Rossendorf, Bautzner Landstraße 400, 01328 Dresden, Germany

**Keywords:** Superconducting properties and materials, Surfaces, interfaces and thin films, Structural properties, Computational science

## Abstract

Striving to improve the critical current density *Jc* of superconducting YBa_2_Cu_3_O_6+x_ (YBCO) thin films via enhanced vortex pinning, the interplay between film growth mechanisms and the formation of nanosized defects, both natural and artificial, is systematically studied in undoped and BaZrO_3_ (BZO)-doped YBCO thin films. The films were grown via pulsed laser deposition (PLD), varying the crystal grain size of the targets in addition to the dopant content. The microstructure of the PLD target has been observed to have a great impact on that of the deposited thin films, including the formation of vortex pinning centers, which has direct implications on the superconducting performance, especially on the isotropy of flux pinning properties. Based on experimentally measured angular dependencies of *Jc*, coupled with a molecular dynamics (MD) simulation of flux pinning in the YBCO films, we present a quantitative model of how the splay and fragmentation of BZO nanorods artifically introduced into the YBCO film matrix explain the majority of the observed critical current anisotropy.

## Introduction

To obtain the freedom to engineer future high-temperature superconductor (HTS) applications for desired operating magnetic field and temperature ranges, it is necessary to optimize the vortex pinning landscape for an enhanced, isotropic flux pinning performance^1–6^. In addition to naturally formed crystalline defects, which typically have spatial dimensions distinctly below the superconducting coherence length, defect-engineering with artificially produced pinning centers (APCs) with dimensionalities of 1D–3D have been observed to be extremely effective^7–10^. However, the complex nucleation process of YBCO during PLD process, that leads to growth island size variation, and the manner in which this could affect the size and distribution of the nanoscale structural defects is chiefly neglected. Especially, a clear gap exists in the current literature regarding how ordered arrays of nanoscale defects can also influence and regulate the distribution and growth of more effective APCs and thus decrease the anisotropy by allowing vortices to be trapped in a wider angular range^11^. Partly, the clear lack of information on the subject is arguably be due to the rather general assumption that during PLD process, the film growth method of our choice, the target material is largely decomposed on the atomic level, and thus its properties should not have an effect on the formation and nucleation of particles on the substrate surface. This assumption, which our studies have led us to challenge, would precariously force one to downplay the potential importance of target microstructure on the functional properties of derived films.

The angular dependence of the *J*_c_ has an excellent physical importance providing an approach to the problem of flux pinning and vortex dynamics anisotropy in HTSs, both from the experimental and theoretical point of view. For instance, in the angular dependent critical current plots, one can easily observe how the various types of pinning centers such as correlated linear, columnar or planar defects and, on the other hand, defects based on growth mechanisms together with YBCO’s intrinsic pinning can dramatically alter the angular dependence of *J*_c_(*B*)^4^. For understanding the origin of angular dependent flux pinning *J*_c_(*θ*), experimental tools like transmission electron microscopy (TEM) are often exploited to probe the structural properties and features, such as the defects naturally formed during the film growth, as well as the size, shape, orientation and distribution of the artificially produced and self-assembled pinning centers^5,12,13^. However, methods like positron annihilation spectroscopy, which gives information about vacancy-type defects, are only rarely utilized on HTS thin films^14,15^. In addition to experimental scrutiny, simulations offer a way of understanding the roles that different pinning landscapes may have in explaining the angular and magnetic field dependencies of *J*_c_, as well as a method for designing the most effective pinning centers for future applications^16–18^.

In this work, we experimentally demonstrate the often downplayed effects of PLD target synthesis method and crystallinity on the growth kinetics and nucleation of particles and the distribution of chemical elements in YBCO thin films. The original used targets for depositing films differ from each other by the average crystal grain size, that is, nanocrystalline (n-YBCO) and microcrystalline (*μ*-YBCO). The phenomenon is widely investigated structurally, magnetically and resistively, and discussed together with the results of theoretical simulations in order to form a comprehensive picture of how the anisotropies of different kinds of pinning sites result in complicated angular dependent flux pinning behaviour.

## Results and Discussion

### Microstructure and defect formation

The microstructure and defect formation of the films is investigated by atomic force microscope (AFM) and TEM. Based on the detailed surface microstructure analysis shown in Supplementary Information (SI), we can conclude that the average in-plane surface particle diameter for the undoped n-YBCO is clearly smaller than that for the undoped *μ*-YBCO films. This indicates a greater number of nucleation sites and smaller growth island size in n-YBCO films which, on the other hand, cause improved flux pinning by defects localized on boudaries between single particles fused together and on the contact points of the growth island network when the film is formed with the Volmer-Weber growth mode^19,20^.

**Figure 1:**
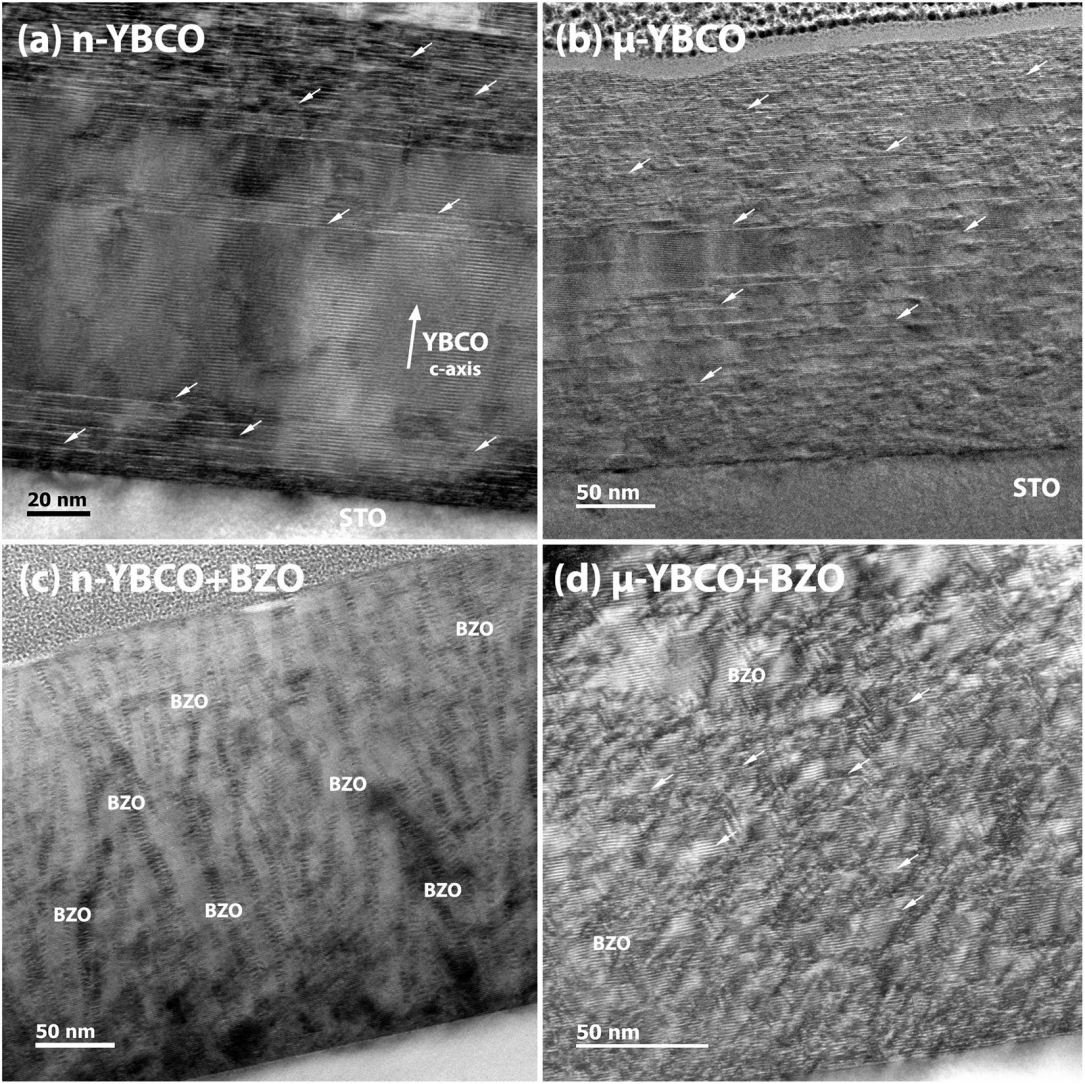
Cross-sectional TEM images of both undoped and BZO doped YBCO films deposited from targets with varying grain sizes (**a**–**d**). The bright vector arrows depict stacking faults. Extended BZO nanocolumns (labeled) are observed in n-YBCO + BZO (**c**), whereas broken BZO nanorods and short stacking faults are seen in *μ*-YBCO + BZO (**d**).

As shown in the cross-sectional TEM images in Fig. 1a, the n-YBCO exhibits the presence of long stacking faults in the bottom, middle and top layers along with the twin boundaries, whereas in *μ*-YBCO (Fig. 1b), the short stacking faults and twin boundaries (see SI) are observed throughout the film. The presence of a large number of short stacking faults throughout the *μ*-YBCO film could be related to the relaxation of strain, as revealed by decreased inhomogeneous strain *ε*_WH_ explained in crystallographic properties of SI.

Comparing the BZO doped n- and *μ*-YBCO, Fig. 1c,d, the stacking faults are only visible in *μ*-film and they are relatively short and randomly distributed. The diameters of BZO nanorods in the films grown using n- and *μ*-grain sized targets average at 7.5 nm and 6.5 nm, respectively, whereas the average distances between nanorods are estimated to be 9 nm and 12 nm, respectively. In the n-films, the BZO rods seem to be unbroken and relatively long, with smaller splay (85–203 nm in length and 5–15° tilted) than in the *μ*-film, where they are short and more tilted (21–45 nm in length and 20–25° tilted) as presented in Fig. 1c,d, respectively. This spacing is calculated over several cross-sectional areas. Since we are using these values in simulations, therefore only the average values are reported here. Moreover, for both undoped and BZO doped films, the n-films are slightly thicker (≈15 nm) than the *μ*-films. In Table 1, the collection of naturally and artificially created defects within the undoped and BZO doped n- and *μ*-YBCO films with the details of BZO nanorods are presented.

**Table 1:** A compilation of TEM results depicting the microscopic characterisitics of undoped and BZO doped n- and *μ*-YBCO films.

Samples	Stacking faults	Splay of nanorods (°)	Twin boundaries
*n*-YBCO	Bottom layer with long stacking faults	—	In bottom layer
*μ*-YBCO	Randomly distributed short stacking faults	—	Visible (see SI)
*n*-YBCO + BZO	Few in number	5–15 (unbroken)	Not visible
*μ*-YBCO + BZO	Randomly distributed short stacking faults	20–25 (often broken)	Not visible

Regarding our TEM results (see SI for more details), one is naturally led to wonder what exactly has caused the increased splay and fragmentation of the BZO nanorods in the *μ*-film compared to the n-film. It is known that the tendency of BZO to form long upright nanocolumns is increased as the YBCO unit cell is stretched along its *c*-axis, since a larger YBCO film *c* lattice parameter will better match against unstrained BZO (where *a* = *b* = *c* = 4.193 nm, to be compared with 1/3 of the YBCO *c* parameter)^21^. As shown in SI, the *c*-axis of the doped *μ*-film is indeed very slightly smaller than that of the doped n-film. Furthermore, the n-film may have provided better local conditions for coherent BZO–YBCO interfaces to develop via improved oxygen diffusion through the larger number of grain boundaries and other defects associated with growth island edges^22^. The increased fragmentation of the *μ*-film nanorods is likely to stem from similar origins, but may also have been compounded by the lower local availability of BZO during the growth process. As will be later illustrated in growth mechanism section, we propose that relatively large clusters of crystalline material are transferred as such to the film during n-YBCO deposition, whereas *μ*-YBCO essentially breaks down at the atomic scale, also spreading the BZO components around more evenly and in smaller units.

**Figure 2:**
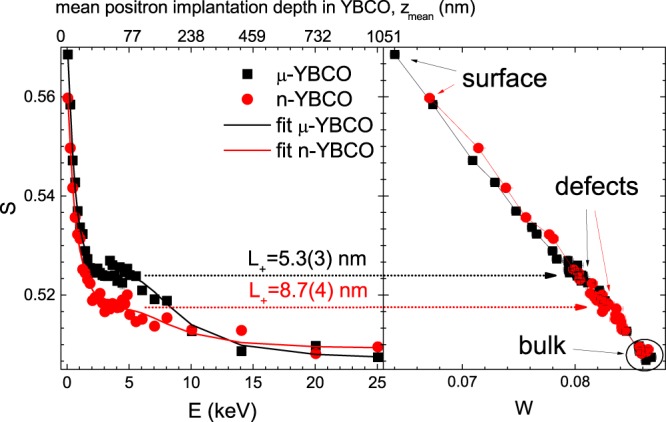
Low electron momentum fraction, *S*, as a function of positron implantation energy, *E* (left panel) and *S* versus the high electron momentum fraction, *W* (right panel). The error bars are about the same size as the symbols. The *S*(*E*) curves have been fitted using VEPFIT code and the thus obtained positron diffusion length, *L*_+_, is given for the *μ*-YBCO and n-YBCO samples.

Variable energy positron annihilation spectroscopy (VEPAS) is used for probing the defect type and concentration in n-YBCO and *μ*-YBCO films. The low electron momentum fraction, *S* (valence electrons) as a function of positron implantation energy, *E*, shown in Fig. 2, is directly proportional to defect concentration and/or defect size. *S* and *W* are unitless and are just a ratio of a certain fraction of the annihilation spectrum (a Gaussian; *S*-in the middle, *W*-on the spectrum tails) normalized to the total number of annihilation events. It can be clearly seen that the *μ*-YBCO sample exhibits a larger concentration of vacancy-like defects such as mono-vacancies or bi-vacancies^23^. The *S*–*W* plot (right panel) shows a linear relation for the most of the data points, which suggests the same defect type across the films’ thickness for both samples and therefore only variations in the defect concentration are expected. Besides this, a sub-surface region, where a line with a slightly different slope can be drawn for a n-YBCO sample, possibly indicates another defect type. In addition, the lower defect concentration in nano-crystalline films is confirmed by the larger positron diffusion length, *L*_+_, which is inversely proportional to the defect concentration. For the analysis of *L*_+_, the VEPFIT code^24^ has been utilized, which permits to fit *S*(*E*) curves for multilayered systems and to acquire the thickness *L*_+_ and specific *S* parameters for each layer within a stack. As derived from VEPFIT analysis of the *S*(*E*) curve the difference in *L*_+_ is close to a factor of 2, which translates to the *μ*-YBCO films having approximately twice the amount of defects over their n-YBCO counterparts.

Since the crystallites size for both film types is much larger than *L*_+_, positrons annihilate mostly in defects situated inside the crystals rather than at grain boundaries (or twin boundaries). The positrons cannot reach the grain boundaries, but are trapped by nearby defects almost upon their implantation. Therefore, the overall localized trapping takes place and most likely positrons preferentially annihilate with open volume defects like dislocation loops and vacancy-like defects. In addition, the defect distribution seems to be constant across the films thickness, which is reflected by a presence of a plateau in *S*(*E*) for 2 keV < *E* < 6 keV.

### Direction dependent superconductivity

Before studying the *J*_c_(*θ*) properties at low temperature, the normal state resistivities were measured at 300 K, giving *ρ* = 192, 175, 166 and 151 *μ*Ω cm for *μ*-YBCO + BZO, *μ*-YBCO, n-YBCO + BZO and n-YBCO, respectively. This is in agreement with the increased number of vacancy type defects observed by VEPAS in films grown from microcrystalline targets, as well as with lowered *T*_c_ values also magnetically obtained for BZO-doped films.

**Figure 3:**
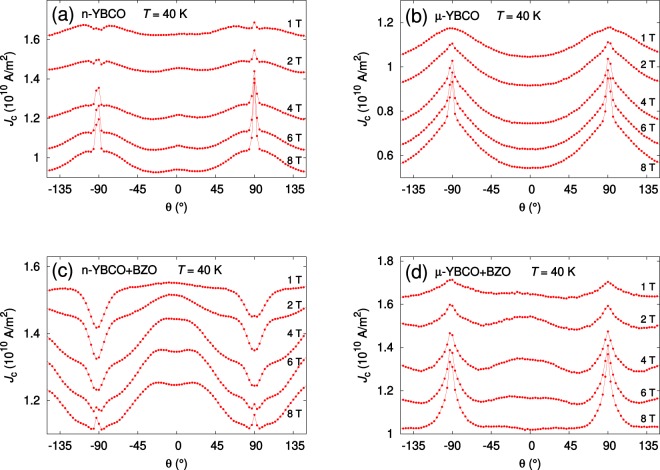
The angular dependencies of *J*_c_ measured at 40 K and different magnetic fields for films deposited from undoped nanocrystalline (**a**) and microcrystalline (**b**) as well as from BZO doped nano- and microcrystalline targets, (**c**) and (**d**), respectively.

In our *J*_c_(*θ*) results, ±90° denotes the direction of *ab*-planes of YBCO, whereas 0° the c-axis of YBCO. A comparison between the *J*_c_(*θ*) of undoped n-YBCO and undoped *μ*-YBCO films (Fig. 3a,b) shows that the n-YBCO curve is much flatter and has a sharp peak at the *B* ||*ab*-direction, whereas the *μ*-YBCO film clearly has broader *ab*-peaks. This isotropic angular dependence of *J*_c_ in n-YBCO is even more pronounced at magnetic fields below 4 T. When looking at the absolute *J*_c_ values, we can see that, as observed in magnetic measurements in the *c*-direction, the undoped n-YBCO has higher *J*_c_ through the whole angular range and in all magnetic fields up to 8 T when compared to undoped *μ*-YBCO films. This is in line with structural results, where a smaller island size produced a larger amount of strain-relaxing structural defects in n-YBCO. In addition, we have observed earlier that the twin domain size is smaller in n-YBCO films which again means the presence of greater number of twin boundaries along the YBCO *c*-axis^25^. This increased number of lattice defects in n-YBCO film is in agreement with the small *c*-peak around 0° (Fig. 3a), which is completely missing in the *μ*-YBCO film. As explained with the vortex path model^26,27^, in *μ*-YBCO film where a great number of short stacking faults occur, vortices have the possibility to be pinned over a shorter distance in the *c*-direction than the standard deviation of the *ab* separation distance of the vortices, in line with the absence of a *c*-axis peak.

In BZO doped YBCO films of Fig. 3c,d, the absolute *J*_c_ value is higher and general anisotropy is smaller in *μ*-YBCO + BZO film measured in fields *B* ≤ 2 T, while the strong *c*-axis peak of n-YBCO + BZO starts to dominate at *B* >2 T, also producing higher *J*_c_ within the whole angular range. In *μ*-YBCO + BZO film, a relatively weak *c*-axis peak in 1 T can be observed, but the *c*-peak is more pronounced in the range 2 T ≤ *B* ≤ 6 T, almost disappearing again in an 8 T field. When compared to n-YBCO + BZO film, the peak of *μ*-YBCO + BZO along the *ab*-plane is relatively broad and it increases with increasing magnetic field. This effective pinning along the *ab*-plane leads to weakened *c*-axis pinning, since the great number of in-plane pinning centers such as stacking faults pin the vortices, especially at high magnetic fields. Similar features in the *J*_c_(*θ*) curve have earlier been observed in BZO doped YBCO films grown at extremely high temperatures, leading to shortened BZO nanocolumns and an increased number of stacking faults parallel to the *ab*-plane^28^. The weakening of the *c*-axis peak as well as the broad *ab*-peak in the *μ*-YBCO + BZO film can be explained by the vortex path model in terms of the trapping angle of the Cu–O spacer layers or stacking faults^26^. In the n-YBCO + BZO film, the *ab*-peak first forms a dip that evolves into a sharp peak with increasing magnetic field above 6 T.

Because of the more important vortex–vortex interactions at high magnetic fields, an ever-increasing number of vortices start to pin along the individual BZO nanorods since the long sideways steps along the *ab*-plane are prohibited^26^. Therefore, we can conclude that the field dependence of *J*_c_(*θ*) in both BZO doped YBCO films can be explained with the vortex path model where, as confirmed by TEM, besides the BZO nanocolumn network, a clearly different natural vortex pinning landscape occurs in n-YBCO + BZO and *μ*-YBCO + BZO.

### Target grain size based growth mechanism

**Figure 4:**
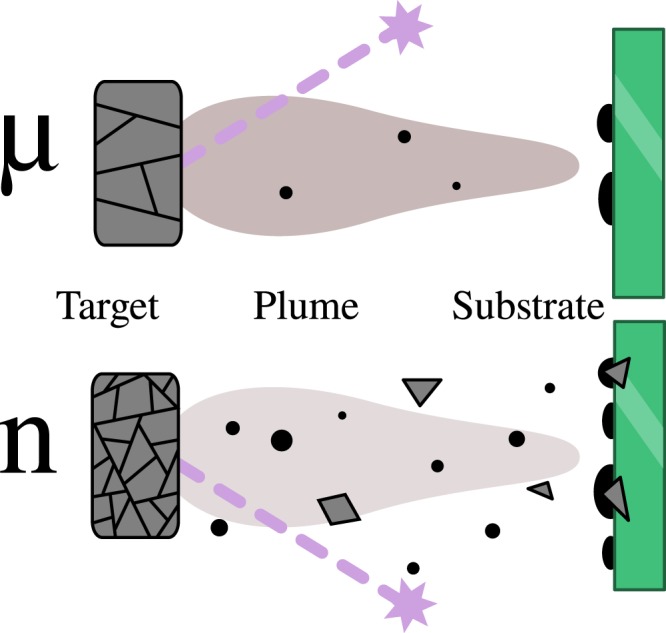
A schematic of the proposed effect of PLD target grain size on the growth process of the deposited film. Upon receiving a laser pulse (stars and dashed lines), the microcrystalline *μ*-YBCO (top) produces a smooth plume of individual atoms and ions (gray cloud) with very few multi-atom clusters (black dots and gray polygons) in comparison with the nanocrystalline n-YBCO (bottom) which has many more grain boundaries over a given ablation surface. Due to their low mobility on the substrate, the larger fragments will more readily act as individual growth centers, leading to the observed increase in film surface granularity when a n-YBCO target is used instead of *μ*-YBCO.

We have a great variety of parameters, such as energy density of the laser and substrate – target distance in the PLD process that need to be optimized. Previous studies^12,29^ showed that these paramters have an impact on the properties of YBCO films but the effect of target grain size has been chiefly neglected. One of the traditional assumptions regarding the PLD process is that the laser breaks down the surface of the target at the atomic level^30^. It is therefore not trivially clear that the grain size of the target should have any effect on the final composition of the deposited film. Our results, in particular the observed angular anisotropy of *J*_c_, do show such a difference, however. We propose to explain this in terms of how the target granularity affects the particle size distribution of the PLD plume. A schematic of the proposed difference between micro- and nanocrystalline YBCO targets is presented in Fig. 4. Due to the larger density of grain boundaries in n-YBCO it seems plausible that the ablation laser could cleave off a proportionally larger number of multi-atom clusters in addition to the single atoms and ions that, on the other hand, dominate the composition of the *μ*-YBCO plume^30^. The larger clusters coming off n-YBCO will have less mobility than individual adatoms on the substrate, resulting in a larger number of individual film growth centers appearing with n-YBCO. As a consequence, we indeed observe almost twice as many growth islands on the surface of a fully-grown n-YBCO film. On the other hand, judging by the consistency of the final film thicknesses, the total amount of matter passed from the target to the substrate does not significantly depend on the target granularity. It certainly takes less energy to cleave off a large cluster of atoms from the target than the same amount of atoms individually, but this can be balanced by the lower probability that a heavy cluster attains and retains enough kinetic energy to reach the substrate. Thus lighter fragments (or individual atoms) receive a proportionally larger kinetic energy per mass than heavier, more voluminous fragments during the ablation process.

**Figure 5:**
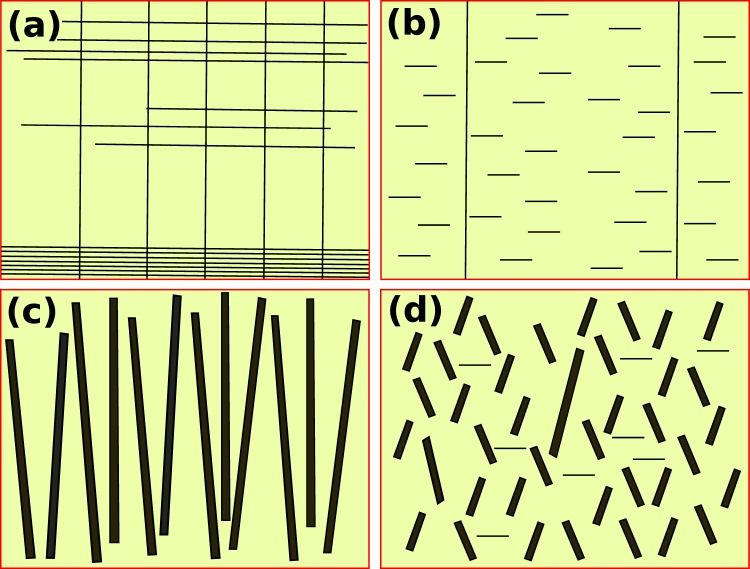
The schematic diagram of the defects grown in undoped n-YBCO (**a**), undoped *μ*-YBCO (**b**), n-YBCO + BZO (**c**) and *μ*-YBCO + BZO (**d**) films during the deposition. Long vertical lines refer to threading dislocations along the *c*-axis whereas both short and long horizontal lines to stacking faults along the *ab*-plane. The thick and variously tilted, both short and extended, columns represent the BZO induced nanorods.

**Figure 6:**
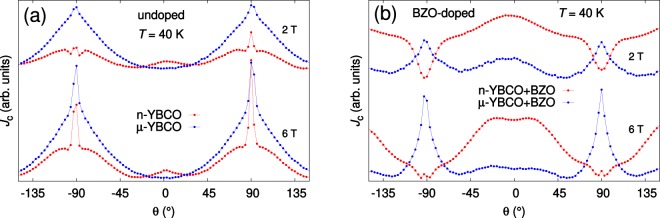
The shape comparison of the angular dependent *J*_c_ (40 K: 2 T and 6 T fields) in undoped (**a**) and BZO doped (**b**) YBsCO films deposited from nanocrystalline and microcrystalline targets.

In Fig. 5, we have presented the schematic of distribution of natural and artificial defects based on our structural, microstructural and flux pinning results. Comparing the undoped cases in Fig. 5a,b, the n-YBCO not only has extended stacking faults at the interface but it also has a high density of threading dislocations, related to the smaller growth islands. The n-YBCO also has relatively long stacking faults in the top layer of the film, increasing the *ab*-plane pinning. Due to the growth of larger growth islands in *μ*-YBCO than in n-YBCO, it turns out that there are not only the short and randomly distributed stacking faults but also it has lesser number of threading dislocations than in n-YBCO as revealed by the c-peak in Fig. 3a. The random growth of short stacking faults is critical, as not only does it hinder the *c*-axis pinning, but also contributes to the broad *ab*-peaks. In the doped cases, as presented in Fig. 5c,d, the stacking faults in n-YBCO + BZO are completely absent, when multidirectionally tilted nanorods were induced. These tilted nanorods can be a source of the double *c*-axis peak shown in Fig. 6b. Moreover, the elongated BZO nanorods passing through the entire film thickness produce a broad and intense *c*-peak because of their both size and shape similarities with the vortices that can effectively pin them even at high fields of 6 T and 8 T as depicted in Fig. 3c. The randomly distributed stacking faults in *μ*-YBCO + BZO can also cause the BZO rods to grow shorter and strongly splayed with unspecified directions. Both randomly distributed and shortened BZO nanorods and stacking faults reduce the *c*-axis vortex pinning as the vortices could take several steps unlike in n-YBCO + BZO, where the vortices are strongly pinned along the elongated nanorods. The short and randomly distributed nanorods contribute to the relatively weak *c*-axis pinning as shown in Fig. 3d only up to 4 T, and strikingly lose their effect at higher fields.

### Nanostructure induced critical current anisotropy

In order to compare the shape and the anisotropy in *J*_c_(*θ*) curves, the most important data is plotted at fields of 2 T and 6 T, as shown in Fig. 6. In Fig. 6, the lowest point for both the data measured at 2 T and 6 T was shifted to the same level to make the shape comparison easier. The formation and the shape evolution of the *c*-axis peak with the effect of multi-structured and broadened peak along the YBCO *ab*-plane as well as their link with the in-plane and out-of-plane structural pinning centers will be discussed in the light of MD simulations.

Firstly, comparing the results of undoped films presented in Fig. 6a, the presence of long stacking faults in n-YBCO makes the in-plane vortex pinning more effective which leads to the sharp and narrow *ab*-peaks. The *ab*-peaks are quite small at 2 T but more intense at 6 T which can be explained by the effect of high density of vortices pinned within stacking faults and CuO_2_ at high fields^28^. It is notable that at both low and high fields, the *ab*-peaks occured as sharp peaks with shoulders around them. The shoulders in the vicinity of the *ab*-peaks in undoped n-YBCO can be due to the interplay of in-plane and out-of-plane correlated pinning, instead of being a sign of any unorthodox pinning at intermediate angles^17^. As explained earlier, the n-YBCO contains more nucleation centers than *μ*-YBCO leading to smaller growth island sizes, which again creates a large amount of *c*-axis oriented dislocation-type pinning centers. These out-of-plane threading dislocations interact with the long in-plane stacking faults thus acting as a source of occurance of the shoulders. The small *c*-peak, present both at low and high fields in n-YBCO, is directly related to the high density of threading dislocations passing through the film as discussed earlier in detail with Fig. 3. On the other hand, the strong and broad *ab*-peaks observed at 2 T in *μ*-YBCO arise due to the small and randomly distributed stacking faults within the film. However, the *ab*-peaks are significantly intense at 6 T, thus indicating more effective vortex pinning by the randomly distributed stacking faults at high fields. Considering the absence of a *c*-axis peak in *μ*-YBCO, the growth of large islands in this case produces a notably smaller number of threading dislocations which, despite pinning the vortices, are not as effective as to show a *c*-axis oriented peak like in n-YBCO, since the occurence of the *c*-peak is only possible when the angular dependent pinning force has a local maximum along the *c*-axis^31^. Although the *μ*-YBCO has twice the number of vacancy-type defects compared to n-YBCO as shown by our VEPAS measurements, these oxygen vacancy complexes are weak pinning centers and do not contribute to significant correlated pinning unlike defects such as dislocations.

Discussing the angular dependent curves for BZO doped films shown in Fig. 6b, the n-YBCO + BZO has huge dips along the *ab*-plane in a 2 T field but small *ab*-peak seems to arise at 6 T. The absence of *ab*-peaks at 2 T could be related to the high density of elongated and well-ordered, largely *c*-axis oriented nanorods, which worsen the *ab*-plane pinning but improve the *c*-axis pinning in such a way that a broad and immensely strong *c*-peak occurs. This would also suppress the *ab*-peak from arising in its usual direction^32^. At 6 T, the *c*-axis pinning weakens which not only narrows the *c*-peak but also allows the small *ab*-peaks to appear. Here, the elongated and well-ordered BZO rods grow due to the absence of stacking faults, unlike in *μ*-YBCO + BZO. The slight splay of BZO nanorods is also a source of the broad *c*-axis peak^5,17^, whereas the better out-of-plane correlation of the unit cells within n-films and the array of structural defects resulting from the island growth mechanism also contribute to the significant *c*-axis pinning^4^. On the other hand, the smaller number of short and randomly distributed stacking faults in *μ*-YBCO + BZO produce a less intense but broad *ab*-peak at 2 T which becomes sharp and intense at 6 T. The random distribution of stacking faults could also affect the growth of BZO nanorods, reducing the *c*-axis pinning both at low and high magnetic fields.

**Figure 7:**
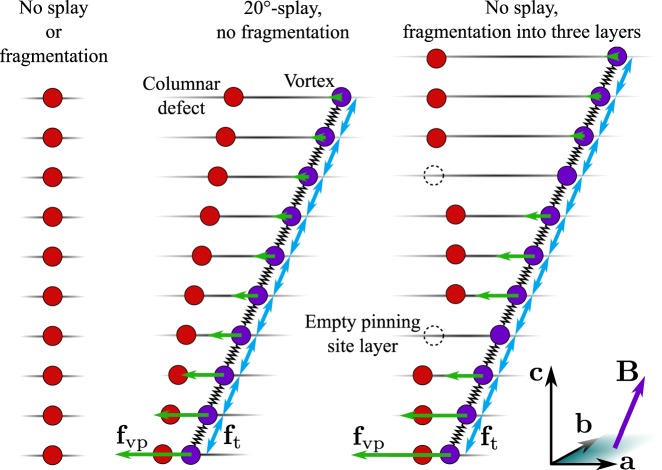
Examples of columnar pinning site models and vortices attracted to them, patterns typically used in the MD simulation of *J*_c_(*θ*) for tilted and fragmented columnar pinning sites. On the left, the simplest case is illustrated where no splay or fragmentation takes place. In the middle, a solid columnar pinning site is tilted 20° from YBCOs *c*-axis shown in the diagram. On the right, no splay takes place but the pinning site is fragmented into three fragment layers with a layer free of pinning sites between them. Such fragment layers were positioned randomly, independent of each other. Notice that in order to keep the thicknesses of the fragmented layers constant, the number of pinning site particles may vary by ±1 as the nanorods are fragmented, shown schematically above. The pinning force **f**_vp_, vortex line tension **f**_t_ and vortex line tension experienced by every vortex particle are also presented.

In order to support this flux pinning mechanism, we have calculated the effect of the tilt angle and length of the artificially produced BZO nanorods using MD-simulations^18^, since the presence of both splay and fragmentation in BZO columnar pinning sites is observed by TEM. In order to study these effects on the critical current angular dependency separately, MD simulation was employed with the pinning site configurations illustrated in Fig. 7. The simulation model is based on a layer structure that restricts the movement of each particle into a specific layer parallel to YBCO’s *ab*-plane. This layer structure limits the angular range of simulations to ±60°, as measured from the YBCO *c*-axis^18^. Columnar pinning sites are modeled by chains of immovable particles spanning several layers, whereas single pinning sites only have one corresponding particle. Vortices are modeled in the same manner, their corresponding particles being allowed to move within their layers. The forces experienced by the vortices in different pinning site configurations, as well as the implementation of the splay and fragmentation of columnar pinning centers are explained in detail in SI.

**Figure 8:**
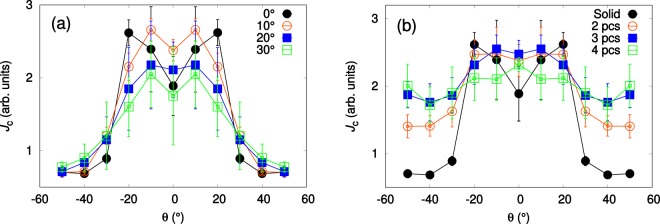
Simulated *J*_c_(*θ*) curves for solid nanorods with 0°, 10°, 20° and 30° of splay (**a**) and 0°-splayed nanorods fragmented into 1, 2, 3 and 4 pieces (**b**), as explained in Fig. 7. The absolute values between (**a**,**b**) are comparable with each other.

The *J*_c_(*θ*) curves simulated using the different tilting angles of the nanorods are presented in Fig. 8a. The overall widths of the simulated peaks seem to be somewhat independent of the splay of the nanorods. Near *θ* = 0°, the peak intensity decreases as splay of the nanorods increases. At higher angles *θ* > 30°, the effect seems to be opposite. Double peaks are observed in every case. Surprisingly, simulations run with 10°-splay produce considerably higher *J*_c_ value at *θ* = 0° compared with simulations using 0°-splay. At 0°-splay, high intensity double *c*-axis peak is observed with maxima at angles *θ* = ±20°. The reason for observing peak maxima at 20° instead of 0° is that at this angle the vortices are optimally oriented in such a way that, (i) the nanorods pinning force still overcomes the magnetic force thus aligning the vortices along the nanorods and pinning them strongly and, (ii) the nanorods are splayed just enough so that they are more likely to come across a pinning center and even get simultaneously entangled into several different nanorods. So, in conclusiion, at lower angles than the angle where the peak maxima occurs, the nanorods are more strongly pinned, but the probability of a vortex coming across a pinning site is much lower. At higher angles than this, the vortices have high probability to come across a pinning site, but the magnetic force overcomes the pinning force and the vortices get only partly pinned thus weakening the total pinning force significantly. This same effect is also behind other observed double peak structures for other simulated *c*-peaks. The *J*_c_(*θ*)-curves simulated using different nanorod fragmentation is presented in Fig. 8b. Fragmentation of nanorods clearly widens the *c*-axis peak. A double peak is observed only for unfragmented simulation due to volume maximizing effect presented before. A random positioning of nanorod fragments into their independent layers destroys the double peak as the vortices can easily bend between these fragmentation layers. The intensity of the *c*-axis peak also remains approximately constant until nanorods are fragmented into four pieces. In general, the fragmentation of nanorods into several pieces seems to result in the increased isotropy of the *J*_c_(*θ*)-curves.

**Figure 9:**
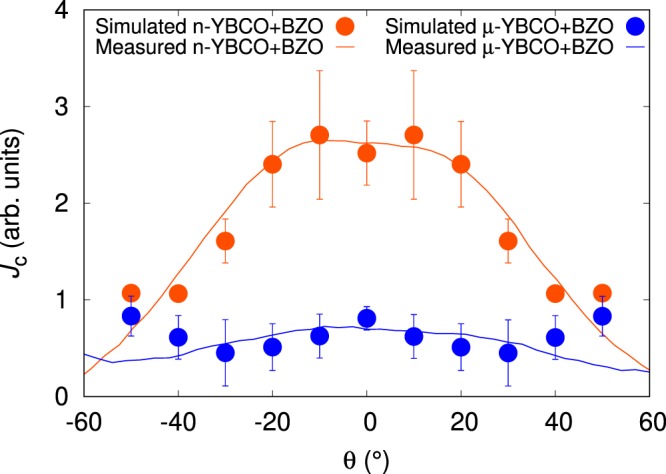
Simulated *J*_c_(*θ*) data together with the experimentally determined *J*_c_(*θ*) curves for *μ*-YBCO + BZO and n-YBCO + BZO films measured in 4 T at 40 K.

In order to mimic the measured properties of *μ*-YBCO + BZO and n-YBCO + BZO films, the simulations with both splay and fragmentation were also carried out. The n-YBCO + BZO sample was modelled with solid 20 particle long columnar pinning sites randomly splayed in 10° angle, as measured by TEM. The *μ*-YBCO + BZO, on the other hand, was modelled with columnar pinning sites that were fragmented into four pieces and using an average splay angle of 22.5°. Figure 9 shows how the *c*-axis peak measured in 4 T and at 40 K fits to the simulated data relatively good. At angles *θ* <± 30°, both simulations reproduce the measured shapes of the peaks indicating such a difference in BZO growth within the n-YBCO and *μ*-YBCO matrices as stated before in the schematic diagram of Fig. 5. However, the experimental data deviates from simulated *J*_c_(*θ*) values at angles *θ* >± 30° due to overly simplified simulation model, where the tilting angles of nanorods and lengths of the fragments are kept constant. Especially, the constant height fragment layers, that are separated by constant distance, create additional symmetry to the system which is definitely not present in reality. The presence of such symmetry might have nebulous effects to the simulated *J*_c_(*θ*)-curves which indeed can be the reason behind observed deviation between measured and simulated data. The effect of the anisotropy of the surrounding YBCO lattice, which is assumed ideal in the simulation model, should also be noted. As a conclusion, we have gained multiple lines of corroborating evidence that the splay and the fragmentation of the BZO nanorods are mainly responsible for the observed critical current and its anisotropy, at least around the YBCO *c*-axis.

## Conclusion

In this work, a great variety of modern techniques in the fields of experimental and computational condensed matter physics were combined to systematically investigate the influence of growth mechanisms and defect formation, both natural and artificial, on the superconducting properties of undoped and BZO doped YBCO thin films. In particular, focus was given to differentiating and modeling how various types of nanosized structural defects can act as vortex pinning centers, enhancing the in-field critical current density and modifying its anisotropy. The results were qualitatively discussed in terms of existing theory, in addition to which the most critical defect types were successfully modeled by a quantitative MD simulation configured based on experimental transmission electron microscopy observations. Collecting the newly obtained critical current anisotropy data and all accessible structural properties of the superconductor materials under our focus, we were able to propose a schematic model that provides a basic framework for vortex pinning landscape engineering. This leads us further closer to the superconducting industrial applications required for the devices needed to be operated within wide temperature, angular and field ranges.

## Methods

### Target synthesis

Four ceramic YBCO samples were synthesized as precursors to the films studied in this work. The samples differ from each other by the average crystal grain size (being nano- or microcystalline) or BZO doping (0% or 4% by mass), so that all combinations of the two parameters are manifested in the sample set. In the following text, we will prefix the grain size class and suffix the doping class to each sample identifier, so that *μ*-YBCO and n-YBCO refer to the undoped micro- and nanocrystalline samples, respectively, and *μ*-YBCO + BZO and n-YBCO + BZO to the corresponding doped samples. The microcrystalline samples *μ*-YBCO and *μ*-YBCO + BZO were manufactured in the form of sintered pellets by the standard solid state ceramic method^33^. We followed the recipe found in ref.^33^. for YBa_2_Cu_3_O_7_ with the exception that the sintering temperature was lowered to 920 °C to avoid the observed melting of one of the reagents or intermediate products at the suggested temperature of 950 °C. The BZO doping was implemented simply by adding stoichiometric amounts of BaCO_3_ and Y_2_O_3_-stabilized ZrO_2_ to the YBCO precursor mix. Heating rates were kept at 100 °C/h and after each burn the furnace was simply switched off to allow passive air cooling back to room temperature. X-ray diffractometry (XRD) and Rietveld refinement showed the samples to be ≥99% phase pure, with crystallite sizes in the order of micrometers (too large for reliable analysis using the Scherrer formula^34^). The relative densities of *μ*-YBCO and *μ*-YBCO + BZO were 76% and 80%, respectively, based on mechanical measurements. The citric acid combustion variant of the sol-gel method^35^ was used to synthesize the nanocrystalline pellet samples n-YBCO and n-YBCO + BZO. The details of the process have been described in our earlier publications^36,37^. The relative densities of n-YBCO and n-YBCO + BZO in the final pelletized form were 86% and 75%, respectively, and the grain sizes of both were ca. 60 nm based on the Scherrer formula^34^. XRD and Rietveld analysis showed no impurity phases, again indicating purities of ≥99%.

### Thin films fabrication

The undoped and BZO doped YBCO films were prepared by pulsed laser deposition (PLD) on (100) SrTiO_3_ (STO) substrates using a 308 nm XeCl excimer laser with a pulse duration of 25 ns and a repetition rate of 5 Hz with a laser fluence of 1.3 Jcm^−2^. The flowing oxygen pressure in the chamber was 0.2 torr and the substrate temperature during the deposition was 750 °C. To get the optimized properties of the films, *in situ* post-annealing treatments were carried out at the annealing temperature *T*_a_ = 725 °C in atmospheric pressure oxygen with heating and cooling rates of 25°/min.

### Structural characterization

The crystallographic properties of the films were determined by x-ray diffraction (XRD) measurements with a Philips X’Pert Pro MPD system (Cu K_*α*_ radiation). To determine the phase purity of the films, (*θ*, 2*θ*) scans in the (00*l*) direction were made. The lattice parameters were determined from detailed 2D (*ϕ*, 2*θ*) texture scans of the YBCO (212)/(122) peaks using 2D Levenberg–Marquardt fitting^38^ of Gaussian peaks. The out-of-plane crystallographic texture was determined by XRD rocking curves (RC) of the YBCO (005) peaks (*ω* scans). The oxygen stoichiometries of the films were estimated from the intensity ratios of the (005)/(007) peaks^39,40^. The correlation length of the lattice was determined from the rocking curve of a (005) peak as *r*_*c*_ = *c*/(*πl*Δ*ω*), where *c* is the longest lattice parameter of YBCO and *l* is the order of the Bragg reflection^41^. The microstrain in *c* direction was determined with the Williamson–Hall method^41^. The FWHM of the (00*l*) peaks was used to determine the microstrain, *ε*_WH_, which in this case describes the variation of the *c* parameter throughout the whole film thickness.

### Surface microstructure and TEM measurements

The surface microstructure and the thickness of the films ≈260 nm for undoped and ≈240 nm for BZO doped films, respectively, were performed on a Bruker Innova AFM. Bright-field transmission electron microscopy (BF-TEM) was performed using a Cs-corrected JEOL JEM 2200FS instrument, operated at 200 kV. Samples for BF-TEM were prepared by cutting a cross-sectional lamella via the Focused Ion Beam (FIB) technique in a FEI Nova 600 Nanolab Dual Beam FIB-SEM. The lamella were extracted using the *in situ* lift out procedure with an Omniprobe extraction needle^42^.

### VEPAS measurements

VEPAS measurements have been conducted at the apparatus for *in situ* defect analysis (AIDA)^15^ of the slow positron beamline (SPONSOR)^14^. Positrons have been implanted into a sample with discrete kinetic energies *E* in the range between 0.05 and 35 keV, which allows for depth profiling from the surface down to a few micrometers. A mean positron implantation depth can be approximated by a simple material density dependent formula: *z*_*mean*_ = 5.71 × *E*^1.62^. Implanted into a solid positrons lose their kinetic energy due to thermalization and after a short diffusion annihilate at delocalized lattice sites or localize in the vacancy like defects and interfaces, usually emitting two anti-collinear 511 keV gamma photons once they meet electrons. Since at the annihilation site thermalized positrons have very small momenta compared to the electrons, a broadening of the 511 keV line is observed mostly due to momentum of the electrons, which is measured with one or two high-purity Ge detectors (energy resolution of of 1.09 ± 0.01 keV at 511 keV). This broadening is characterized by two distinct parameters *S* and *W* defined as a fraction of the annihilation line in the middle (511 ± 0.93 keV) and outer regions (508.56 ± 0.35 keV and 513.44 ± 0.35 keV), respectively. The *S* parameter is a fraction of positrons annihilating with low momentum valence electrons and represents vacancy type defects and their concentration. The *W* parameter approximates overlap of positron wavefunction with high momentum core electrons. Plotting calculated *S* as a function of positron implantation energy, *S*(*E*), provides depth dependent information, whereas *S*–*W* plots are used to examine atomic surrounding of the defect site and its size (type)^43^.

### Magnetic and transport measurements

Magnetic measurements were made with a Quantum Design physical property measurement (PPMS) system, and the onset critical temperatures, *T*_c_, were determined with ac magnetization measurements in the range of 10–100 K (in ac field of 0.1 mT). The critical current densities, *J*_c_, at 10 K were determined from the hysteresis loops using the Bean model for rectangular films: *J*c = 2Δ*m*/[*a*(1 − *a*/3*b*)*V*], where *a* and *b* (*b* ≥ *a*) are the width and the length of the sample, *V* is the sample volume and Δ*m* is the opening of the hysteresis loop^44^. The transport properties of all the films were measured using the horizontal rotator option available for the PPMS. The measurements were done at magnetic fields of 0.5 T, 1 T, 2 T, 4 T, 6 T and 8 T and temperatures at 10 K, 40 K and 77 K with 0° to 360° angular range using 3° of steps. For this purpose, all the films were patterned by wet chemical etching. The etched patterns were 50 *μ*m wide current stripes on each film. The contacts on the films were made by aluminium wire using a TPT HB05 Wire Bonder and without any metal contact pad in-between the bond and surface of YBCO. Based on our earlier studies and on the measurements in this work, the magnetic field dependencies of *J*_c_ as well as the shape of the angular dependence of *J*_c_ are similar at different measurement temperatures below ≈70 K^10,45^. Although the measurements were carried out in a rather wide temperature range, we decided to concentrate the analysis on measurements at 40 K, which is far enough from *T*_c_ and, on the other hand, meets the temperature requirements for power technology applications^1^.

### MD simulations

The angular dependent MD simulations were used to reproduce the shape of *J*_c_(*θ*) around the *B*||*c* of YBCO, by simulating the vortices in the superconducting thin films when different kinds of artificially produced pinning centers are available in the YBCO matrix. The details of the MD simulations are presented elsewhere^18^.

## Supplementary information

Supplementary Information
