# Supplementary material for: Enhanced flux pinning isotropy by tuned nanosized defect network in superconducting YBa2Cu3O6+x films
Source: Sci Rep. 2019 Oct 28;9:15425. doi: 10.1038/s41598-019-51978-0 (PMC6817878; doi:10.1038/s41598-019-51978-0)
Supplement: Supplementary file 1 — Supplementary Information [file 41598_2019_51978_MOESM1_ESM.pdf]

Supplementary Information: Enhanced flux pinning isotropy by tuned nanosized defect network in superconducting $YBa_2Cu_3O_{6+x}$ films

Mukarram Zaman Khan[1,2,*], Elmeri Rivasto[1,2], Jussi Tikkanen[1], Hannes Rijckaert[3], Mika Malmivirta[1], Maciej Oskar Liedke[4], Maik Butterling[4], Andreas Wagner[4], Hannu Huhtinen[1], Isabel Van Driessche[3], and Petriina Paturi[1]

[1]Wihuri Physical Laboratory, Department of Physics and Astronomy, University of Turku, FI-20014 Turku, Finland
[2]University of Turku Graduate School (UTUGS), University of Turku, FI-20014 Turku, Finland
[3]SCRiPTS, Department of Chemistry, Ghent University, Krijgslaan 281 S3, 9000 Ghent, Belgium
[4]Institute of Radiation Physics, Helmholtz-Zentrum Dresden - Rossendorf, Bautzner Landstraße 400, 01328 Dresden, Germany
[*]mukarram.z.khan@utu.fi

Detailed TEM analysis

For making the comprehensive comprasions of the as induced natural and artificial defects in all the films, the TEM images with the resolutions of 20 nm are also presented in Fig. S1. These images are well in line to those in Fig. 1.

Scanning transmission electron microscopy (STEM) was utilized to compare the stacking faults within the undoped n-YBCO and μ-YBCO as presented in Fig. S2. In relation to Fig. 1 in the main manuscript, it can be seen here also (at the resolution level of 100 nm) that the stacking faults are longer in n-YBCO as compared to μ-YBCO where they are observed to be shorter and randomly distributed within the entire film. Moreover, BF-TEM images presented in Fig. S3 clearly reveal the presence of twin boundaries within the undoped n-YBCO and μ-YBCO films.

Crystallographical properties

The 2θ X-ray diffractograms seen in Fig. S4 reflect the good chemical purity and desired growth orientation of the YBCO films. Only YBCO $(00l)$, STO $(h00)$ and, in the samples doped with $BaZrO_3$ (BZO), BZO (200) reflections are observed, i.e. YBCO has grown epitaxially, with its crystallographic c-axis normal to the STO substrate as expected, and no impurity phases need to be considered to explain the data. The BZO dopant also shows cube-on-cube orientation alongside the YBCO matrix, an expected feature due to the self-assembled columnar growth mode of BZO in this system[S1]. Granted, the only visible Bragg reflection (200) is among the most intense ones of BZO, but in the powdered sample approximation an even stronger (by a factor of ~ 3) (110) reflection should be visible at $2\theta = 30.08^\circ$ in superposition with YBCO (004), something our relative intensity measurements on the YBCO (400) and (500) reflections (see Table S1) rule out. This favours the topotaxial growth interpretation.

The detailed 2θ–ϕ scans of the YBCO peak group (212)/(122) shown in Fig. S5 give a graphical comparison of the crystal quality between the four film samples. Biaxial twinning, with a/b twin boundaries running along [110] and $[1\bar{1}0]$, is evident in all films, though an increased ab-plane splay in the BZO-doped ones somewhat obscures the twin structure. The n-films originating from nanocrystalline PLD targets both show slightly better peak definition than their "μ" counterparts (even disregarding the fact that the μ-YBCO film was accidentally attached to the sample holder at a slight tilt, leading to an artificial correlation between 2θ and ϕ), indicating the crystal structure is coherent over longer distances in the n-films.

That the YBCO crystal structure is more homogeneous in the n-films is corroborated by the findings on the YBCO (005) reflection presented in Table S1. In particular, the FWHM ω values show that n-YBCO has by far the best c-axis orientation among the films, whereas n-YBCO+BZO beats μ-YBCO+BZO by a relatively smaller margin in this sense. The FWHM 2θ values reflect a known difference in how the BZO-doped and undoped films handle the elastic stress from the substrate interface: the doped films develop low angle grain boundaries in the ab-plane, nucleating these defects at the BZO impurities, whereas the undoped films prefer clean biaxial twinning and also retain a higher inhomogeneous strain (ε_{WH} in Table S1), as determined by Williamson-Hall (WH) plots[S2,S3]. The addition of BZO also seems to reduce the orthorhombicity of YBCO, suggesting that BZO works as a mild reductant in the system and lowers the oxygen content of YBCO when other synthesis parameters are kept constant[S4].

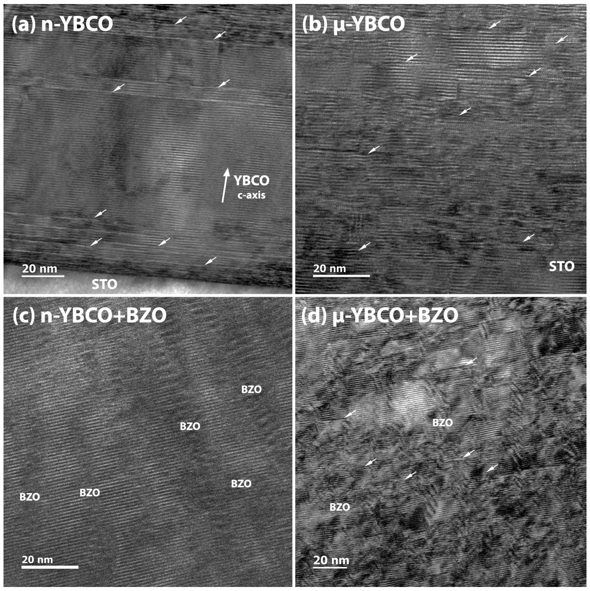

Figure S1. TEM images (20 nm resolution) of the all the films deposited from targets with varying grain sizes (a)−(d). Stacking faults are marked by the bright vectors, whereas the nanorods induced in the doped films are labelled as BZO.

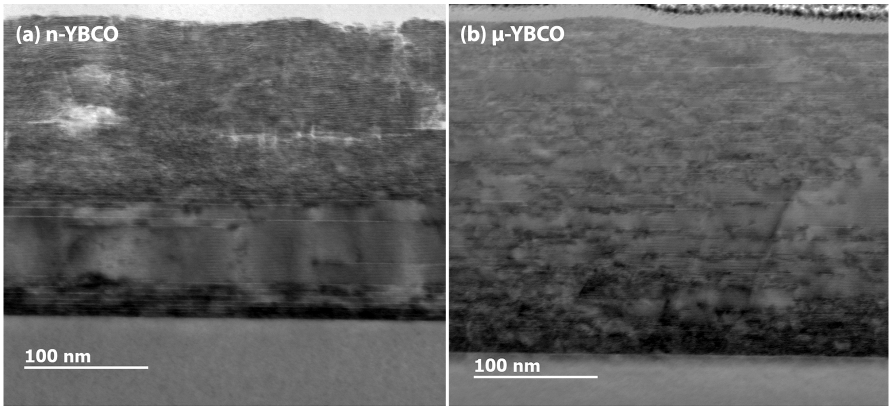

Figure S2. STEM images depicting the comparison of stacking faults with respect to their density and sizes for undoped n-YBCO and μ-YBCO films (a)−(b).

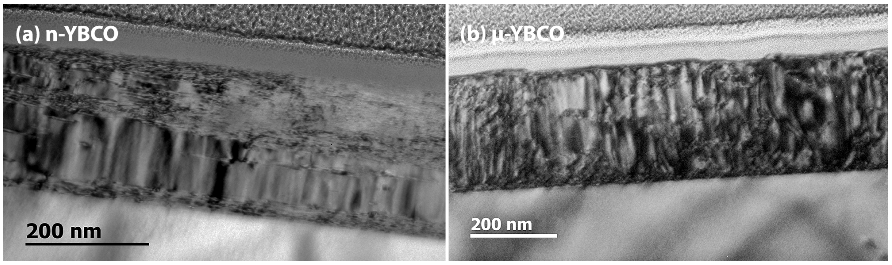

Figure S3. BF-TEM images, displaying the presence of twin boundaries, and taken with a diffraction vector **g** = (100) of undoped n-YBCO and μ-YBCO films (a)−(b).

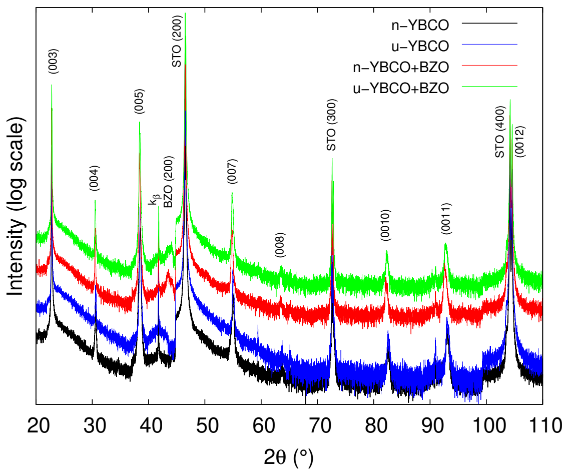

Figure S4. The room temperature X-ray 2θ diffractograms of the undoped and BZO doped YBCO films deposited from nanocrystalline and microcrystalline targets.

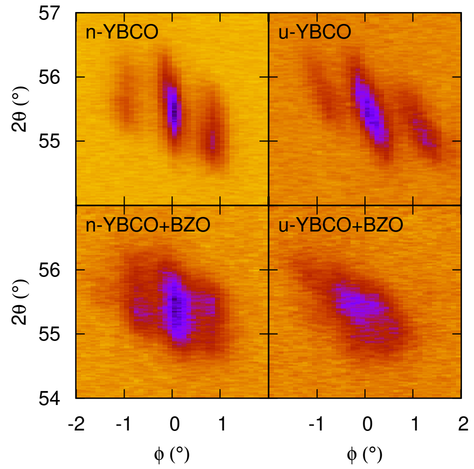

Figure S5. XRD (212)/(122) peaks as a function of 2θ and relative ϕ with normalized intensities for undoped and BZO-doped YBCO films deposited using nanocrystalline and microcrystalline targets.

Table S1. Structural parameters determined from XRD measurements for undoped and BZO doped YBCO films deposited from different kinds of targets.

Samples	YBCO lattice a (Å)	b (Å)	c (Å)	Orthorhombicity (%)	ε_{WH} (%)	FWHM 2θ (005) (°)	FWHM ϕ (102) (°)	FWHM ω (005) (°)	r_c (nm)
n-YBCO	3.831	3.897	11.685	1.710	0.013	0.17	1.30	0.21	19.7
μ-YBCO	3.826	3.893	11.678	1.723	0.008	0.14	1.37	0.29	14.3
n-YBCO+BZO	3.843	3.879	11.722	0.949	0.008	0.11	1.59	0.28	14.8
μ-YBCO+BZO	3.851	3.887	11.716	0.934	0.011	0.13	1.11	0.32	13.1

Surface microstructure

As can be seen from the $2 \times 2\,\mu m^2$ AFM images in Fig. S6, the average in-plane surface particle diameter of ≈ 180 nm for the undoped n-YBCO film is clearly smaller than that of ≈ 300 nm for the undoped μ-YBCO film. The higher out-of-plane scale bar in μ-YBCO film also indicates larger growth islands and thus rougher surface, which is confirmed by the root-mean-square roughness values of 3.3 nm for n-YBCO and 4.8 nm for μ-YBCO film, respectively. To further investigate the film growth and thus the epitaxial crystallization of the YBCO film on STO substrate, we applied only fifteen pulses with the normal laser fluence in the PLD process and studied the grouping and nucleation of three dimensional clusters on a substrate in both undoped n-YBCO and μ-YBCO cases. Although the threshold energy for depositing single YBCO particles at the normal deposition distance between the target and the substrate has been observed to be clearly smaller for n-YBCO than for μ-YBCO targets[S5,S6], we could not find a difference in fragment size at the beginning of the growth process when the nucleation of the particles had already started on the surface of the heated substrate. The in-plane fragment size for both targets was on average $120-200$ nm, which is roughly the same as the island size in the finished n-YBCO film but only half of that in μ-YBCO film.

Magnetic properties

The normalized magnetizations of all four samples are shown as functions of temperature in the main panel of Fig. S7. It can be seen that in undoped YBCO films T_c is on average 5 K higher than that of BZO doped films. However, the films prepared from nanocrystalline target have approximately 1 K higher T_c although the transitions are slightly broader than in films grown from microcrystalline targets. This is in line with VEPAS measurements, where a greater number of vacancy-type defects, for example oxygen vacancy complexes, have been observed. The J_c curves measured at 10 K and calculated using the Bean model[S7] for all undoped and BZO doped YBCO films are shown in the inset of Fig. S7. In terms of vortex pinning, two completely different $J_c(B)$ curve shapes can be observed in undoped and BZO doped YBCO films. First of all, the low field J_c is clearly higher in undoped YBCO films. This can be understood by the volume of BZO rods as well as by the distorted YBCO with lower T_c around the rods that decreases the amount of superconducting material in doped films[S8]. In undoped YBCO films,

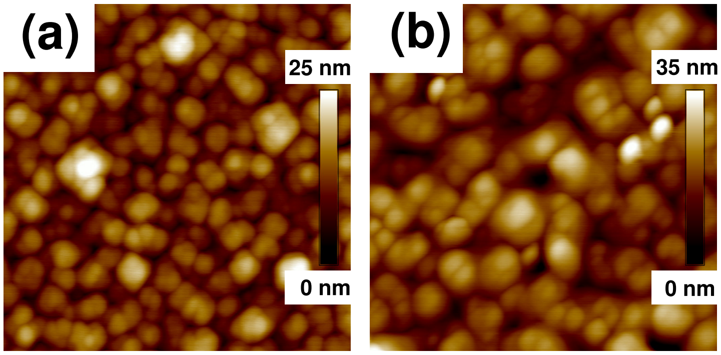

Figure S6. AFM images of the undoped n-YBCO (a) and undoped μ-YBCO (b) films. The scan sizes of the images are $2 \times 2\ \mu m^2$ and the values of the height scale bars are given in subfigures.

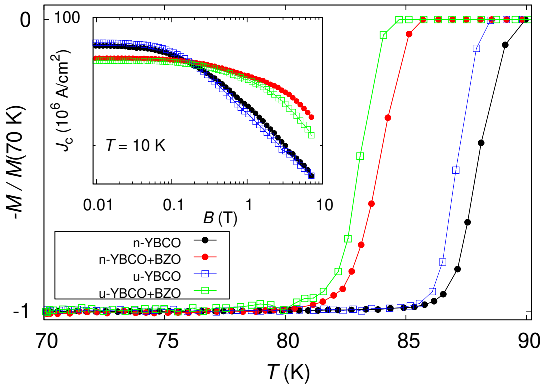

Figure S7. The normalized ac magnetizations as functions of temperature (main panel) and the magnetic field dependencies of J_c determined from the hysteresis loops at 10 K (inset) for undoped and BZO doped YBCO films deposited using nanocrystalline and microcrystalline targets.

the J_c clearly starts to drop below 100 mT while in BZO doped films, before reduction, a longer constant value in J_c curve can be observed. The upper limit of low-field plateau of J_c(B) calculated by criterion $J_c(B)/J_c(0) = 0.9$ is called as accomodation field B^*[S9]. Since the single vortex pinning is important below the B^* and above B^* the collective pinning dominates[S10], we can conclude that the clearly higher J_c above 500 mT is related to columnar network of BZO nanorods formed in the YBCO matrix. In addition to the high density of pinning sites, the characteristic round shape of the J_c curve in BZO doped YBCO is based on the large size of pinning centers. This has been observed to increase the depth of the pinning potential, number of pinned vortices per rod and to suppress the long-range ordering of the vortex lattice[S11]. It is also noteworthy that in both undoped and BZO doped cases, the n-YBCO films have slightly higher J_c above 200 mT when compared with μ-YBCO films, indicating more anisotropic pinning centers and thus the better in-field performance in films grown from nanocrystalline targets.

MD simulations

The MD-simulations, mainly the forces experienced by the vortices in different pinning site configurations as well as the splay and the fragmentation of columnar pinning centers, are implemented as follows. The force between a vortex and pinning site particle depends on the radius of the pinning site, chosen to be 4 nm, which corresponds to measured values presented above. The only force acting across layers is the spring-like line tension force between two vortex particles in adjacent layers, which keeps the vortices structured. Other forces applied in the simulation are the repulsion force between vortices, a drag force, the Lorentz force caused by a current through the sample and an angle dependent magnetic force that acts to align the vortex particles parallel to the external magnetic field. The value of the critical current was solved iteratively by the bisection method where the current was adjusted until certain vortex stability requirements were fulfilled. All the simulations were run on five randomly generated pinning site configurations corresponding to certain splay or fragmentation of the pinning sites

and the external magnetic field was varied between the angles of -50° and 50°, with 10° steps. In every simulation, we used a computationally efficient 200 × 200 unit sized grid with 32 randomly generated columnar pinning sites that had the minimum distance of 26 units from each another. The simulations were run with 14 vortices, corresponding to a magnetic field of 0.75 T. The final $J_c(\theta)$ was determined by the average value of the results of these individual simulations corresponding to the absolute values of a certain angle as well as the standard deviation that is used as an error limit.

The effect of the splay of the columnar pinning sites on the angular dependency of J_c was studied by creating 20-particles-long solid rows of pinning site particles and tilting them to 0°, 10°, 20° and 30° angles. The locations and orientations of the tilted pinning sites were randomly generated. The effect of fragmentation was studied by creating 0°-splay pinning sites that were cut into two, three and four pieces respectively, separated by one completely pinning site particle free layer. The number of particles representing a single pinning site was kept as close to 20 as possible, resulting in 20, 10+10=20, 7+7+7=21 and 5+5+5+5=20 particles per single pinning site, corresponding fragmentation into one, two, three and four pieces, respectively. Pinning site fragments in a single fragment layer, that is fragments confined between layers free of pinning site particles, were randomly positioned into their corresponding layers. Due to empty layers between the fragments, the number of used layers and length of the vortices increased as a number of fragments resulting at worst for three extra layers and vortex particles per vortex. The effect of these extra layers or one extra pinning site particle to $J_c(\theta)$ can be assumed to be negligible, which allows us to compare obtained $J_c(\theta)$ curves between different fragmentations.

References

S1. Baca, F. J. *et al.* Interactive growth effects of rare-earth nanoparticles on nanorod formation in $YBa_2Cu_3O_x$ thin films. *Adv. Funct. Mater.* **23**, 4826–4831 (2013).

S2. Zak, A. K., Majid, W. A., Abrishami, M. & Yousefi, R. X-ray analysis of ZnO nanoparticles by williamson–hall and size–strain plot methods. *Solid State Sci.* **13**, 251 (2011).

S3. Mele, P. *et al.* Ultra-high flux pinning properties of $BaMO_3$-doped $YBa_2Cu_3O_{7-x}$thin films (m = zr, sn). *Supercond. Sci. Technol.* **21**, 032002 (2008).

S4. Farneth, W., Bordia, R., McCarron, E., Crawford, M. & Flippen, R. Influence of oxygen stoichiometry on the structure and superconducting transition temperature of $YBa_2Cu_3O_x$. *Solid State Commun.* **66**, 953 (1988).

S5. Huhtinen, H., Laiho, R. & Paturi, P. Superconductivity in low-dimensional structures. *Phys. Low-Dimensional Struct.* **11/12**, 93 (1998).

S6. Huhtinen, H., Järvinen, J., Laiho, R., Paturi, P. & Raittila, J. Laser deposition from a nanostructured YBaCuO target: Analysis of the plume and growth kinetics of particles on $SrTiO_3$. *J. Appl. Phys.* **90**, 1521–1528 (2001).

S7. Wiesinger, H. P., Sauerzopf, F. M. & Weber, H. W. On the calculation of J_c from magnetization measurements on superconductors. *Phys. C* **203**, 121–128 (1992).

S8. Peurla, M., Huhtinen, H., Tse, Y. Y., Raittila, J. & Paturi, P. Structural properties of YBCO thin films deposited from different kinds of targets. *IEEE T. Appl. Supercond.* **17**, 3608–3611 (2007).

S9. Cai, C., Holzapfel, B., Hänisch, J., Fernandez, L. & Schultz, L. Magnetotransport and flux pinning characterictics in RBaCuO (R=Gd,Eu,Nd) and $(Gd_{1/3}Eu_{1/3}Nd_{1/3})$BaCuO high-$T_c$ superconducting thin films on $SrTiO_3$. *Phys. Rev. B* **69**, 104531 (2004).

S10. Krusin-Elbaum, L., Civale, L., Thompson, J. R. & Feild, C. Accommodation of vortices to columnar defects: Evidence for large entropic reduction of vortex localization. *Phys. Rev. B* **53**, 11744 (1996).

S11. Palonen, H., Jäykkä, J. & Paturi, P. Modeling reduced field dependence of critical current density in $YBa_2Cu_3O_{6+x}$ films with nanorods. *Phys. Rev. B* **85**, 024510 (2012).
